# Low-Dose Methotrexate and Bone Health: Pathophysiological and Clinical Perspectives

**DOI:** 10.3390/jcm15052013

**Published:** 2026-03-06

**Authors:** Anton Sokhan, Judith Haschka, Zora Messner, Jochen Zwerina, Roland Kocijan

**Affiliations:** 1 1st Medical Department, Ludwig Boltzmann Institute of Osteology at Hanusch Hospital of OEGK and AUVA Trauma Centre Meidling, Hanusch Hospital, 1140 Vienna, Austria; anton.sokhan@lbg.ac.at (A.S.); judith.haschka@lbg.ac.at (J.H.); jochen.zwerina@lbg.ac.at (J.Z.); 2 Medizinische Abteilung, Universitätsklinikum St. Pölten, 3100 St. Pölten, Austria; zora.messner@gmail.com; 3 Metabolic Bone Diseases Unit, School of Medicine, Sigmund Freud University Vienna, 1020 Vienna, Austria

**Keywords:** methotrexate, fracture, osteoblast, osteoclast, osteopathy

## Abstract

This narrative review summarizes current evidence on the molecular and cellular effects of low-dose methotrexate (LD-MTX) on bone tissue. In addition, it critically examines the limited and heterogeneous data on LD-MTX-associated osteopathy, a rare and incompletely understood condition that may be underrecognized in clinical practice. Finally, the review highlights key knowledge gaps and outlines future research directions aimed at improving diagnosis, management, and prevention. In total, 451 relevant articles were retrieved, and 71 studies were included in our review. Methotrexate (MTX) has been shown to prevent bone loss associated with inflammatory rheumatic diseases, primarily through its anti-inflammatory properties. However, current evidence highlights a variety of negative effects on bone associated with LD-MTX therapy, including osteoblast dysfunction, increased osteoclastogenesis, and endothelial damage. Collectively, these effects may result in deterioration of microarchitecture, impaired bone healing and insufficiency fractures. Despite the long and successful use of MTX in rheumatology, our knowledge of its effects on bone and awareness of LD-MTX osteopathy remain limited, potentially leading to delayed or missed diagnoses. Recent clinical studies highlight the potential underestimation of this condition and emphasize the need for further research to establish clear diagnostic criteria and treatment guidelines, as well as to achieve a more comprehensive understanding of the complex pathophysiology underlying LD-MTX osteopathy.

## 1. Introduction

Originally developed in the 1940s as a folate antagonist, methotrexate (MTX) is still commonly used at high-doses (≥500 mg/m^2^) in the chemotherapy of malignant tumors in children and adults [1,2,3,4,5] and at low-doses (5–25 mg per week) as first-line therapy in the treatment of rheumatoid arthritis (RA), systemic lupus erythematosus, Crohn’s disease, vasculitis, atopic dermatitis, psoriasis (arthritis), and other inflammatory arthropathies for decades [6,7]. A detailed review of the effects of high-dose methotrexate (HD-MTX) on bone tissue has recently been published [8]. In this narrative review, we summarize and analyze existing data on the effects of low-dose methotrexate (LD-MTX), within the dosage ranges used in clinical rheumatology practice, on bone tissue.

LD-MTX is the most commonly used medication in rheumatology practice because it has been demonstrated to be an effective and safe agent [9]. The immunemodulating mode of action of LD-MTX is complex and may be anti-inflammatory rather than antiproliferative [10,11,12]. It alters T-cell expression of cytokines and adhesion molecules [13,14,15,16]. Consequently, it significantly decreases predominant expression of pro-inflammatory cytokines such as IL-1, IL-6, or TNF-α [10,17,18,19,20] and increases the gene expression of anti-inflammatory cytokines IL-4 and IL-10, resulting in anti-inflammatory effects. Moreover, LD-MTX reduces IL-1β-induced PGE2 production [21] by inhibiting cyclooxygenase 2 (COX-2) synthesis, neutrophil chemotaxis [10,21,22], and by inducing synovial cells apoptosis [23,24]. Several other pathways have been suggested over the last years; however, they are not the topic of this review.

Besides the well-known positive effects of LD-MTX, hematologic, skin-related, gastrointestinal, hepatic, infectious, and pulmonary adverse effects have been reported [25]. The effects of LD-MTX on bone are less pronounced and remain poorly studied. Inflammatory rheumatic diseases (IRDs) by themselves are associated with systemic bone loss, osteoporosis, and low-traumatic fractures, caused by inflammation, auto-immunity, and co-factors such as disease activity and glucocorticoid treatment [26]. Thus, there is strong evidence of a causal link between inflammation and bone loss in IRDs [10,26,27,28,29]. It is generally accepted that LD-MTX prevents bone loss associated with IRDs, primarily through its anti-inflammatory properties. However, LD-MTX may also have negative effects on bone tissue in some cases. Moreover, clinical case reports and case series indicated, that long-term LD-MTX therapy may potentially lead to the development of LD-MTX osteopathy (LD-MTX-O), mainly in weight-bearing bones, characterized by insufficiency fractures and pain [30,31,32,33].

Current literature highlights a significant gap in understanding the effects of long-term LD-MTX treatment on bone tissue. The lack of clear diagnostic criteria, as well as insufficient awareness of LD-MTX-O, is especially challenging. In the present review, we summarize current knowledge on the potential positive and negative effects of LD-MTX on bone tissue in general and discuss the clinical manifestations of LD-MTX-O in particular.

## 2. Materials and Methods

**Figure 1:**
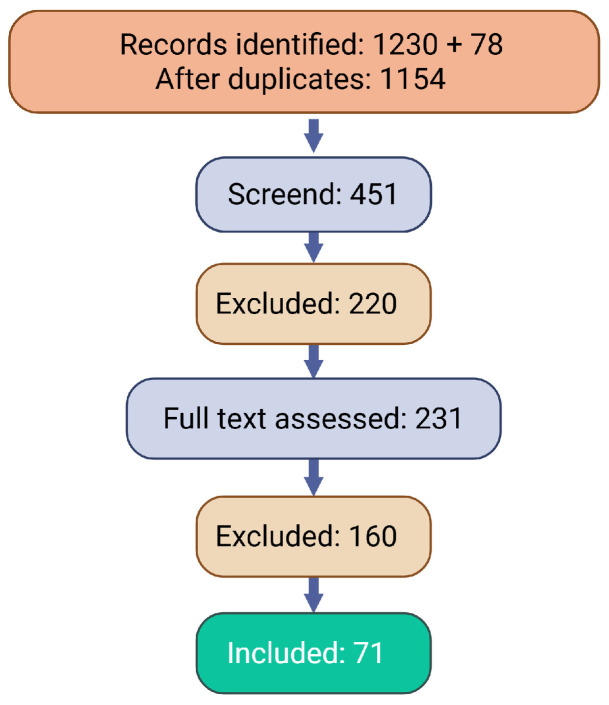
The diagram summarizes the identification, screening, eligibility assessment, and inclusion of studies in the narrative synthesis: 1230 records were identified through database searches and 78 via reference lists; after the removal of duplicates, 451 records were screened by title and abstract, 231 full-text articles were assessed for eligibility, and 71 studies were included in the final synthesis.

We conducted a literature search of the PubMed, Scopus, and Web of Science databases (up to 1 December 2025) using terms including “methotrexate,” “bone,” “osteopathy,” “fracture,” “osteoblast,” “osteoclast,” “osteocyte,” “chondrocyte,” and “bone marrow,” supplemented by manual screening of reference lists from key publications. We obtained 451 relevant articles, which were independently reviewed by two authors to verify eligibility. After excluding studies that did not meet the inclusion criteria, 71 studies were included in our review. The inclusion criteria were studies reporting the effects of LD-MTX on bone, as well as the clinical manifestations, diagnosis, and treatment of LD-MTX-induced osteopathy. The diagram summarizes the identification, screening, eligibility assessment, and inclusion of studies in the narrative synthesis presented in Figure 1.

## 3. Potential Beneficial Effects of LD-MTX on Bone Health

The bone-protective effects of LD-MTX might be mainly explained by controlling systemic inflammation. The mechanism of the anti-inflammatory effect of LD-MTX has been confirmed by a series of in vitro and animal studies. Reduction in pro-inflammatory cytokines such as TNF-α, IL-1 and IL-6 attenuates osteoclast differentiation and activity, thereby limiting inflammation-driven bone erosion [34]. LD-MTX inhibits T-cells and fibroblast-like synoviocytes (FLS) by suppressing nuclear factor-κB (NF-κB), an important factor that promotes cell proliferation and inflammation [35,36], and also osteoclastogenesis. This results in a reduction in the overall number of osteoclasts in the eroded joint [37,38,39], in primary spongiosa [40], and in bone marrow [41], paralleled by a significant reduction in bone resorption area [42] in a dose-dependent manner [39,43]. Furthermore, an additional direct effect of MTX on osteoclast precursor cells [42], including suppression of upregulated osteoclastogenesis by downregulating RANKL and RANK [37,39,42,43] and osteopontin production [44] without changes in the expression of Osteoprotegerin (OPG), has been observed [42]. Consequently, a significant decrease in the RANKL/OPG ratio can be found [42].

The bone-protective effects of LD-MTX in patients with IRDs have been confirmed by multiple studies in humans. Treatment with LD-MTX has been shown to reduce bone resorption activity in patients with IRDs. This effect is evidenced by a decrease in deoxypyridinoline levels and an increase in bone alkaline phosphatase levels [45]. However, other authors found no significant differences between baseline and later measurements for osteocalcin, bone alkaline phosphatase, or deoxypyridinoline [46].

Understanding of bone changes in patients treated with LD-MTX is limited due to a lack of data from histomorphometric analysis obtained from patient bone biopsies. To date, only Minaur et al. have published a histomorphometric report on the effects of LD-MTX on trabecular bone, based on iliac crest bone biopsies taken from four premenopausal women with recent-onset RA (<12 months) before they started LD-MTX and again after one year of treatment [46]. They found no adverse effects on bone mineral density (BMD) or on any measured histomorphometric parameters, including cancellous bone area, osteoid perimeter, osteoid width, mean wall width, resorption cavity indices, mineral apposition rate and mineralizing perimeter. Additionally, no significant negative effects of eight weeks of LD-MTX treatment on tibial bone microarchitecture (thickness of the cortical bone, vascularity, size of the cells, number of cells, matrix homogeneity, arrangement of osteons, and periosteal irregularity) or tendon morphology in animal models have been observed [47]. Furthermore, an experimental rat model study found no negative effects on endochondral fracture healing, including the formation of new periosteal and medullary bone, as well as chondroid tissue [48].

BMD assessment using two-dimensional dual-energy X-ray absorptiometry (DXA) technique in female RA patients after long-term LD-MTX therapy showed no negative effect on lumbar spine and hip BMD [49,50,51]; furthermore, no bone loss was observed in children with juvenile rheumatoid arthritis [52]. It was also shown that LD-MTX monotherapy does not increase the risk of vertebral, hip, forearm, or humerus fractures in a US-wide observational RA cohort [53]. A population-based Norway study found that LD-MTX users with RA had a fracture risk comparable to that of participants with RA overall [54]. In adjuvant arthritis animal models, LD-MTX treatment maintained an age-dependent increase in lumbar and femoral BMD [55], attenuated bone loss in the tibia [56], and increased periarticular BMD at the femur [41].

Thus, MTX has been shown to prevent bone loss associated with IRDs, primarily through its anti-inflammatory action [41,42,55,56]. It is effective in reducing inflammation in arthritis [38,56], and direct positive effects on bone have been observed.

## 4. Potential Detrimental Effects of LD-MTX on Bone Health

Early in vitro studies showed that an LD-MTX inhibits osteoblastic cell proliferation in a dose-dependent manner, without affecting basal osteoblastic phenotypic expression [57] or the proliferative potential of human bone-derived cells [6,58]. LD-MTX had no influence on either the function or proliferation of mature osteoblasts [58]. A more recent study revealed that MTX concentrations more than one hundredfold lower than those measured in patients receiving LD-MTX still exert significant inhibitory effects on osteoblast proliferation and mitochondrial metabolism [59]. It has also been shown that long-term LD-MTX treatment significantly stimulates osteoblast apoptosis [60] and reduces osteoblast proliferation, bone surface density [10,60,61], and metabolic activity [60]. Moreover, it increases monocytic cell apoptosis and reduces their growth [10]. These effects are accompanied by increased osteoclast formation and activity [40,61]. Other authors found that LD-MTX inhibits the proliferation, viability, and mitochondrial activity of endothelial cells [62,63,64], suggesting that such anti-angiogenic effects can negatively impact bone tissue healing and the osteointegration of dental implants [59,62]. Moreover, LD-MTX does not change the adipogenic differentiation potential and did not alter peroxisome proliferator-activated receptor gamma (PPARγ) expression, but it decreases preadipocyte proliferation, promotes the hypertrophic growth of adipocytes, and reduces fibroblast proliferation [65].

Fan et al., using young growing rats, found that long-term use of LD-MTX reduces primary bone formation, as evidenced by decreased primary spongiosa height and bone volume [40]. This was associated with increased osteoclast formation and bone surface density, as well as a decreased osteoblast bone surface density in the primary spongiosa.

Liu et al. investigated the effect of different anti-inflammatory medications on bone in healthy adult Sprague–Dawley rats [66]. LD-MTX over 12 weeks caused a significant reduction in trabecular bone volume/tissue volume (BV/TV), trabecular number (TbN), and increased trabecular separation in the proximal tibial metaphysis, with increased osteoclast surface (OcS) and osteoclast surface/bone surface OcS/BS, and decreased femoral but not lumbar vertebral BMD. Such changes reduced the energy to maximum load and energy to fracture load of the femur, as measured by the three-point bending test [66].

**Figure 2:**
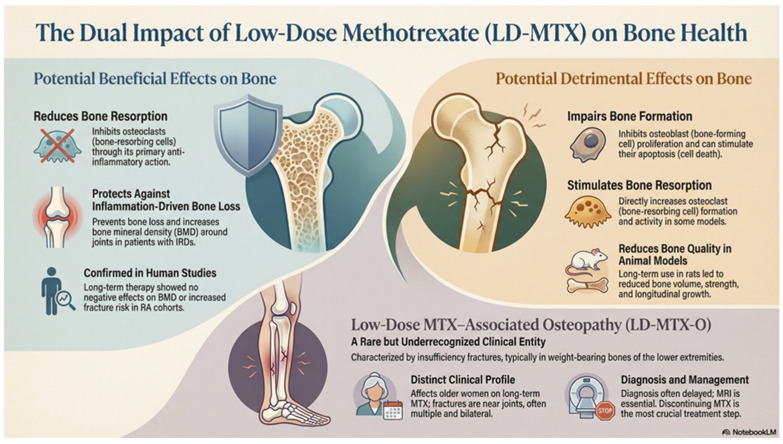
Overview of potential beneficial and detrimental effects of low-dose MTX treatment on bone metabolism in chronic inflammatory diseases, including low-dose MTX-osteopathy (The figure was generated using the AI tool GPT-4 “NotebookLM in Pro” based on reviewed articles).

Thus, available data confirm that, despite the clear therapeutic benefits of LD-MTX in the treatment of IRDs, its use might be associated with multiple alterations that may significantly impair bone homeostasis in some cases. Especially in the absence of inflammatory state, methotrexate potentially exerts different and detrimental effects on bone. In summary, LD-MTX was reported to reduce osteoblast proliferation and metabolic activity, decrease fibroblast proliferation, stimulate osteoclastogenesis, and inhibit endothelial cell proliferation, thereby impairing bone healing. The current data indicate a significant gap in our understanding of the effects of long-term LD-MTX on bone and underscore the critical importance of further studies addressing dosage and potential negative long-term effects of LD-MTX exposure on bone metabolism. An overview of the effects of LD-MTX on bone based on reviewed articles is shown in Figure 2.

## 5. Low-Dose Methotrexate-Associated Osteopathy (LD-MTX-O): A Special and Rare Clinical Entity

LD-MTX-O represents a rare but likely underrecognized clinical entity. It is characterized by insufficiency fractures that occur almost exclusively in weight-bearing bones of the lower extremities, with pain being the leading symptom. Affected patients are predominantly women receiving methotrexate therapy for inflammatory rheumatic diseases. At present, neither diagnostic nor classification criteria have been established. Given the overall beneficial effects of methotrexate on bone metabolism and the lack of convincing evidence linking low-dose methotrexate to osteoporosis or typical osteoporotic fractures, the existence of a distinct LD-MTX-O has been questioned or alternatively considered an exceedingly rare complication. This uncertainty is further reinforced by the very limited body of evidence, as, up to 2022, only 32 publications reporting a total of 80 adult patients with stress fractures attributed to LD-MTX-O had been published. [31] However, recent clinical studies have reported notable numbers of LD-MTX-associated fractures: 83 patients were identified between 2018 and 2022 at a single outpatient clinic in Hamburg [67], 33 patients between 2019 and 2021 at an outpatient clinic in Edinburgh [68], and 92 patients in a French multicenter retrospective study conducted between 2012 and 2024 [69]. These findings suggest that LD-MTX-O is likely a far more significant and underestimated clinical problem than previously recognized.

Available clinical data show that demographic and clinical characteristics of patients with LD-MTX-O are similar across published case series, with a predominance of postmenopausal women with RA and years of successful LD-MTX treatment [31,67,68,69,70]. MTX dosages ranged between 15 and 25 mg per week, and the duration of MTX treatment was >3 years in most cases [31,33,71]. This suggests that the condition is more likely the result of an individual reaction to the drug rather than dose-dependent toxicity [72]. Over the past decades, numerous studies have been conducted to determine the impact of genetic polymorphisms on the development of MTX adverse effects. Urano et al. showed that methylenetetrahydrofolate reductase (MTHFR) gene polymorphism in a cohort of women with RA in Japan did not appear to be a clinically useful marker for predicting fracture risk, whereas LD-MTX use was independently associated with the incidence of nonvertebral fractures [32]. Unfortunately, to our knowledge, this is the only study investigating the influence of genetic polymorphisms on bone tissue properties in patients with IRDs.

The main clinical manifestation of such fractures is chronic, load-dependent pain in the weight-bearing lower extremity, typically occurring in the absence of adequate trauma and accompanied by minimal local soft-tissue edema [31,33,67,68,69]. Cases of osteonecrosis of the jaw associated with LD-MTX have also been reported [73,74,75]. The delay in diagnosing such fractures is dramatic. The reported average period from the first symptoms to diagnosis ranged from 7 to 17 months, resulting in long-term pain and disability [33,67,68,69]. This delay in diagnosis may be due to relatively mild local symptoms that mimic arthritis, and X-rays are insufficient to diagnose LD-MTX-O, especially in the early stages. Thus, in 2025, Hauser et al. reported that only one-third of cases were diagnosed using plain radiographs [68]. Therefore, early use of more sensitive imaging techniques such as computed tomography (CT), scintigraphy, or preferably magnetic resonance imaging (MRI) may reduce the delay in diagnosis [67,68,69,71].

The fracture distribution in these patient cohorts differs fundamentally from that typically observed in osteoporotic fractures, as more than 70% of LD-MTX-treated patients develop insufficiency fractures involving the upper or lower ankle joint, and nearly two-thirds present with fractures of the bones forming the knee. This accumulation clearly indicates the primary manifestation of LD-MTX-O close to a joint, in the metaphyseal area of weight-bearing bones of the lower extremity [67,68,69]. Moreover, up to 85% of patients have more than one fracture, and up to 84% of patients have bilateral fractures [68,69,71]. In all published case series, MRI findings showed band-shaped insufficiency fractures without bone deformities in the metaphyseal area, along with bone marrow edema and sclerosis paralleling the former provisional zones of calcification and growth plates [33,69,70,71,76]. Nevertheless, it remains unclear whether the changes visible on CT or MRI are truly specific to MTX-O.

Despite the fact that osteoporosis is associated with insufficiency fractures, only 50–75% of the patients with LD-MTX-O were diagnosed with osteoporosis [33,68,69,71]. These findings underscore the need for a thorough evaluation to diagnose LD-MTX-O, even in the absence of osteoporosis.

Currently, there are no clear data on changes in laboratory parameters reflecting bone metabolism in patients with LD-MTX-O, due to the limited number of studies and conflicting results. Some studies with larger patient groups reported that levels of calcium, 25(OH)vitamin D, alkaline phosphatase phosphate, bone-specific alkaline phosphatase, osteocalcin, procollagen type 1 N-terminal propeptide (PINP), β-crosslinked aminotransferase (β-CT), parathyroid hormone, and deoxypyridinoline (DPD) were within the normal range [67,68]. In contrast, Rolvien T. et al. (2021) found elevated bone-specific alkaline phosphatase levels, low osteocalcin levels, and elevated bone resorption parameters (i.e., DPD crosslinks in urine) [33].

There is a huge gap in understanding bone changes in LD-MTX-O due to the lack of data on histomorphometric analysis obtained from patient bone biopsies. We found only two studies examining bone histological parameters in patients with LD-MTX-O. Histomorphometry of transiliac bone biopsies of two postmenopausal female patients with LD-MTX-O showed a low bone turnover state, reduced osteoid matrix formation with reduction in osteoblast surfaces, osteoid matrix parameters, and tetracycline labeling, indicating osteoblast inhibition [77]. Bone biopsies obtained from fracture sites of four patients with LD-MTX-O showed that local bone microstructure was characterized by an unchanged BV/TV ratio and a higher trabecular number but a lower trabecular thickness. Furthermore, again a low number of osteoblasts but a high number of osteoclasts were detected, leading to a lower osteoblast surface and a higher osteoclast surface compared to those of the control. Backscattered electron imaging confirmed highly prevalent eroded surfaces but an overall similar bone mineral density distribution. Additional histomorphometric analysis of three fracture biopsies from the distal tibiae and the calcaneus revealed the presence of fracture calluses and woven bone but no detection of osteonecrosis. This was associated with a generally high bone turnover [33].

The role of glucocorticoids in LD-MTX-O remains unclear. In the described case series, approximately half of the patients had not received glucocorticoids in the last three years [31,67,68]. Patients receiving LD-MTX and steroids showed more limited mobility in the event of a fracture, but the changes in bone metabolism were independent of steroid use [67]. In contrast to methotrexate, biological agents such as TNF-α inhibitors and IL-6 receptor antagonists (e.g., tocilizumab) are well-established to exert protective effects on bone metabolism and bone mineral density [53,78,79]. To date, neither an increased fracture risk nor the occurrence of insufficiency fractures comparable to those reported with MTX has been described.

Data on potential therapeutic strategies are currently scarce. MTX discontinuation appears to be crucial in the treatment of LD-MTX-O. Discontinuation of MTX is associated with greater clinical improvement in pain and weight-bearing capacity during fracture healing [67,68,69]. Patients who continued MTX had a significantly higher incidence of further fractures compared to those who stopped MTX [68]. Thus, the earlier use of the most informative diagnostic methods, such as MRI and scintigraphy, to reduce diagnostic delays appears to be one of the key factors in reducing the severity of osteopathy and lowering the number of subsequent fractures.

Similar to insufficiency fractures of other origins or bone marrow edema, unloading might represent a relevant therapeutic component. However, evidence is lacking. So far, no studies on orthopedic procedures have been published. Adequate vitamin D intake is essential, and the use of specific anti-osteoporotic medications may be considered [33]. Von Brackel et al. reported that osteoanabolic agents significantly improve mobility restrictions [67]. It can be assumed that anti-resorptive medications, such as bisphosphonates or denosumab, may have beneficial effects in the presence of bone marrow edema [70]. The dual-acting sclerostin antibody romosozumab may offer advantages in the treatment of LD-MTX-O due to its anti-resorptive and osteoanabolic effects. However, there is no evidence to support this.

This narrative review was conducted to summarize the available data on both the effects of LD-MTX on bone and the clinical manifestations, diagnosis, treatment, and prevention of LD-MTX-O. Unfortunately, despite its long-term use in clinical practice, there is currently insufficient scientific evidence to clearly and unambiguously establish a causal relationship between the negative effects of LD-MTX on bone and other potential contributing factors, such as IRDs themselves, the use of glucocorticoids, age, and genetic interactions, which would help identify the most important factors driving the development of osteopathy and fractures. The epidemiology, as well as the clear diagnosis and treatment of LD-MTX-O, also remain poorly studied. This knowledge gap requires further research.

## 6. Conclusions

Despite its well-established efficacy in the treatment of inflammatory rheumatic diseases, long-term LD-MTX therapy might have multifactorial effects on bone tissue. The overall impact of LD-MTX on bone remains poorly understood. Although positive effects on bone metabolism have been described, negative effects have also been reported. These effects include osteoblast dysfunction, increased osteoclastogenesis, and endothelial damage. To date, there are no data on its effect on osteocytes. Particularly concerning is the lack of research into the mechanisms underlying severe bone alterations that lead to LD-MTX-O and insufficiency fractures of the weight-bearing bones. A multifactorial pathogenesis is likely, and genetic polymorphisms may represent one plausible explanation. An analysis of the available data reveals a significant gap in both the understanding of its actual prevalence and the existence of clear diagnostic criteria and treatment strategies. This underscores the need for further research to better understand the complex pathophysiology underlying LD-MTX-O and improve approaches for treatment and prevention.

## Data Availability

No new data were created or analyzed in this study.

## Abbreviations

The following abbreviations are used in this manuscript:

LD-MTX	Low-dose methotrexate
HD-MTX	High-dose methotrexate
RA	Rheumatoid arthritis
MTX	Methotrexate
COX-2	Cyclooxygenase 2
IRDs	Inflammatory rheumatic diseases
LD-MTX-O	Low-dose methotrexate osteopathy
FLS	Fibroblast-like synoviocytes
NF-κB	Nuclear factor-κB
RANKL	Receptor activator of nuclear factor kappa-Β ligand
RANK	Receptor activator of nuclear factor kappa-B
OPG	Osteoprotegerin
PPARγ	Peroxisome proliferator-activated receptor gamma
BMD	Bone mineral density
DXA	Dual-energy X-ray absorptiometry
BV/TV	Trabecular bone volume/tissue volume
TbN	Trabecular number
OcS	osteoclast surface
OcS/BS	Osteoclast surface/bone surface
MTHFR	Methylenetetrahydrofolate reductase
CT	Computed tomography
MRI	Magnetic resonance imaging
PINP	Procollagen type 1 N-terminal propeptide
β-CT	β-crosslinked aminotransferase
DPD	Deoxypyridinoline

## References

[1] Paul S., Kantarjian H., Jabbour E.J. (2016). Adult Acute Lymphoblastic Leukemia. Mayo Clin. Proc..

[2] Brown P., Inaba H., Annesley C., Beck J., Colace S., Dallas M., DeSantes K., Kelly K., Kitko C., Lacayo N. (2020). Pediatric Acute Lymphoblastic Leukemia, Version 2.2020, NCCN Clinical Practice Guidelines in Oncology. J. Natl. Compr. Cancer Netw..

[3] Joerger M., Huitema A.D., Illerhaus G., Ferreri A.J. (2012). Rational administration schedule for high-dose methotrexate in patients with primary central nervous system lymphoma. Leuk. Lymphoma.

[4] Christensen A.M., Pauley J.L., Molinelli A.R., Panetta J.C., Ward D.A., Stewart C.F., Hoffman J.M., Howard S.C., Pui C.H., Pappo A.S. (2012). Resumption of high-dose methotrexate after acute kidney injury and glucarpidase use in pediatric oncology patients. Cancer.

[5] Howard S.C., McCormick J., Pui C.H., Buddington R.K., Harvey R.D. (2016). Preventing and Managing Toxicities of High-Dose Methotrexate. Oncologist.

[6] Minaur N.J., Jefferiss C., Bhalla A.K., Beresford J.N. (2002). Methotrexate in the treatment of rheumatoid arthritis. I. In vitro effects on cells of the osteoblast lineage. Rheumatology.

[7] Singh J.A., Saag K.G., Bridges S.L., Akl E.A., Bannuru R.R., Sullivan M.C., Vaysbrot E., McNaughton C., Osani M., Shmerling R.H. (2016). 2015 American College of Rheumatology Guideline for the Treatment of Rheumatoid Arthritis. Arthritis Rheumatol..

[8] Sokhan A., Hartmann M.A., Blouin S., Erben R.G., Zwerina J., Haschka J., Behanova M., Kocijan R. (2026). Effects of high-dose methotrexate on bone metabolism: A narrative literature review. Bone Rep..

[9] Torres R.P., Santos F.P., Branco J.C. (2022). Methotrexate: Implications of pharmacogenetics in the treatment of patients with Rheumatoid Arthritis. ARP Rheumatol..

[10] Cutolo M., Sulli A., Pizzorni C., Seriolo B., Straub R.H. (2001). Anti-inflammatory mechanisms of methotrexate in rheumatoid arthritis. Ann. Rheum. Dis..

[11] Kremer J.M. (1994). The mechanism of action of methotrexate in rheumatoid arthritis: The search continues. J. Rheumatol..

[12] Segal R., Caspi D., Tishler M., Wigler I., Yaron M. (1989). Short term effects of low dose methotrexate on the acute phase reaction in patients with rheumatoid arthritis. J. Rheumatol..

[13] Wessels J.A., Huizinga T.W., Guchelaar H.J. (2008). Recent insights in the pharmacological actions of methotrexate in the treatment of rheumatoid arthritis. Rheumatology.

[14] Constantin A., Loubet-Lescoulie P., Lambert N., Yassine-Diab B., Abbal M., Mazieres B., de Preval C., Cantagrel A. (1998). Antiinflammatory and immunoregulatory action of methotrexate in the treatment of rheumatoid arthritis: Evidence of increased interleukin-4 and interleukin-10 gene expression demonstrated in vitro by competitive reverse transcriptase-polymerase chain reaction. Arthritis Rheum..

[15] Paillot R., Genestier L., Fournel S., Ferraro C., Miossec P., Revillard J.P. (1998). Activation-dependent lymphocyte apoptosis induced by methotrexate. Transplant. Proc..

[16] Genestier L., Paillot R., Fournel S., Ferraro C., Miossec P., Revillard J.P. (1998). Immunosuppressive properties of methotrexate: Apoptosis and clonal deletion of activated peripheral T cells. J. Clin. Investig..

[17] Brody M., Bohm I., Bauer R. (1993). Mechanism of action of methotrexate: Experimental evidence that methotrexate blocks the binding of interleukin 1 beta to the interleukin 1 receptor on target cells. Eur. J. Clin. Chem. Clin. Biochem..

[18] Novaes G.S., Mello S.B., Laurindo I.M., Cossermelli W. (1996). Low dose methotrexate decreases intraarticular prostaglandin and interleukin 1 levels in antigen induced arthritis in rabbits. J. Rheumatol..

[19] Dolhain R.J., Tak P.P., Dijkmans B.A., De Kuiper P., Breedveld F.C., Miltenburg A.M. (1998). Methotrexate reduces inflammatory cell numbers, expression of monokines and of adhesion molecules in synovial tissue of patients with rheumatoid arthritis. Br. J. Rheumatol..

[20] Smith-Oliver T., Noel L.S., Stimpson S.S., Yarnall D.P., Connolly K.M. (1993). Elevated levels of TNF in the joints of adjuvant arthritic rats. Cytokine.

[21] Vergne P., Liagre B., Bertin P., Cook-Moreau J., Treves R., Beneytout J.L., Rigaud M. (1998). Methotrexate and cyclooxygenase metabolism in cultured human rheumatoid synoviocytes. J. Rheumatol..

[22] Kraan M.C., de Koster B.M., Elferink J.G., Post W.J., Breedveld F.C., Tak P.P. (2000). Inhibition of neutrophil migration soon after initiation of treatment with leflunomide or methotrexate in patients with rheumatoid arthritis: Findings in a prospective, randomized, double-blind clinical trial in fifteen patients. Arthritis Rheum..

[23] Nakazawa F., Matsuno H., Yudoh K., Katayama R., Sawai T., Uzuki M., Kimura T. (2001). Methotrexate inhibits rheumatoid synovitis by inducing apoptosis. J. Rheumatol..

[24] Lee S.Y., Park S.H., Lee S.W., Lee S.H., Son M.K., Choi Y.H., Chung W.T., Yoo Y.H. (2014). Synoviocyte apoptosis may differentiate responder and non-responder patients to methotrexate treatment in rheumatoid arthritis. Arch. Pharm. Res..

[25] Solomon D.H., Glynn R.J., Karlson E.W., Lu F., Corrigan C., Colls J., Xu C., MacFadyen J., Barbhaiya M., Berliner N. (2020). Adverse Effects of Low-Dose Methotrexate: A Randomized Trial. Ann. Intern. Med..

[26] Coury F., Peyruchaud O., Machuca-Gayet I. (2019). Osteoimmunology of Bone Loss in Inflammatory Rheumatic Diseases. Front. Immunol..

[27] Haugeberg G., Conaghan P.G., Quinn M., Emery P. (2009). Bone loss in patients with active early rheumatoid arthritis: Infliximab and methotrexate compared with methotrexate treatment alone. Explorative analysis from a 12-month randomised, double-blind, placebo-controlled study. Ann. Rheum. Dis..

[28] Schett G., Gravallese E. (2012). Bone erosion in rheumatoid arthritis: Mechanisms, diagnosis and treatment. Nat. Rev. Rheumatol..

[29] Fardellone P., Salawati E., Le Monnier L., Goeb V. (2020). Bone Loss, Osteoporosis, and Fractures in Patients with Rheumatoid Arthritis: A Review. J. Clin. Med..

[30] Robin F., Cadiou S., Albert J.D., Bart G., Coiffier G., Guggenbuhl P. (2021). Methotrexate osteopathy: Five cases and systematic literature review. Osteoporos. Int..

[31] Ruffer N., Krusche M., Beil F.T., Amling M., Kotter I., Rolvien T. (2022). Clinical features of methotrexate osteopathy in rheumatic musculoskeletal disease: A systematic review. Semin. Arthritis Rheum..

[32] Urano W., Furuya T., Inoue E., Taniguchi A., Urano T., Kotake S., Sekita C., Inoue S., Hara M., Momohara S. (2009). Associations between methotrexate treatment and methylenetetrahydrofolate reductase gene polymorphisms with incident fractures in Japanese female rheumatoid arthritis patients. J. Bone Miner. Metab..

[33] Rolvien T., Jandl N.M., Sturznickel J., Beil F.T., Kotter I., Oheim R., Lohse A.W., Barvencik F., Amling M. (2021). Clinical and Radiological Characterization of Patients with Immobilizing and Progressive Stress Fractures in Methotrexate Osteopathy. Calcif. Tissue Int..

[34] Schett G., Stach C., Zwerina J., Voll R., Manger B. (2008). How antirheumatic drugs protect joints from damage in rheumatoid arthritis. Arthritis Rheum..

[35] Spurlock C.F., Gass H.M., Bryant C.J., Wells B.C., Olsen N.J., Aune T.M. (2015). Methotrexate-mediated inhibition of nuclear factor kappaB activation by distinct pathways in T cells and fibroblast-like synoviocytes. Rheumatology.

[36] Bustamante M.F., Garcia-Carbonell R., Whisenant K.D., Guma M. (2017). Fibroblast-like synoviocyte metabolism in the pathogenesis of rheumatoid arthritis. Arthritis Res. Ther..

[37] Teramachi J., Kukita A., Li Y.J., Ushijima Y., Ohkuma H., Wada N., Watanabe T., Nakamura S., Kukita T. (2011). Adenosine abolishes MTX-induced suppression of osteoclastogenesis and inflammatory bone destruction in adjuvant-induced arthritis. Lab. Investig..

[38] Le Goff B., Soltner E., Charrier C., Maugars Y., Redini F., Heymann D., Berthelot J.M. (2009). A combination of methotrexate and zoledronic acid prevents bone erosions and systemic bone mass loss in collagen induced arthritis. Arthritis Res. Ther..

[39] Suematsu A., Tajiri Y., Nakashima T., Taka J., Ochi S., Oda H., Nakamura K., Tanaka S., Takayanagi H. (2007). Scientific basis for the efficacy of combined use of antirheumatic drugs against bone destruction in rheumatoid arthritis. Mod. Rheumatol..

[40] Fan C., Cool J.C., Scherer M.A., Foster B.K., Shandala T., Tapp H., Xian C.J. (2009). Damaging effects of chronic low-dose methotrexate usage on primary bone formation in young rats and potential protective effects of folinic acid supplementary treatment. Bone.

[41] Suzuki Y., Nakagawa M., Masuda C., Ide M., Uehara R., Ichikawa Y., Mizushima Y. (1997). Short-term low dose methotrexate ameliorates abnormal bone metabolism and bone loss in adjuvant induced arthritis. J. Rheumatol..

[42] Revu S., Neregard P., Klint E.a., Korotkova M., Catrina A.I. (2013). Synovial membrane immunohistology in early-untreated rheumatoid arthritis reveals high expression of catabolic bone markers that is modulated by methotrexate. Arthritis Res. Ther..

[43] Lee C.K., Lee E.Y., Chung S.M., Mun S.H., Yoo B., Moon H.B. (2004). Effects of disease-modifying antirheumatic drugs and antiinflammatory cytokines on human osteoclastogenesis through interaction with receptor activator of nuclear factor kappaB, osteoprotegerin, and receptor activator of nuclear factor kappaB ligand. Arthritis Rheum..

[44] Sun Y., Yao Y., Ding C.Z. (2014). A combination of sinomenine and methotrexate reduces joint damage of collagen induced arthritis in rats by modulating osteoclast-related cytokines. Int. Immunopharmacol..

[45] El Miedany Y.M., Abubakr I.H., El Baddini M. (1998). Effect of low dose methotrexate on markers of bone metabolism in patients with rheumatoid arthritis. J. Rheumatol..

[46] Minaur N.J., Kounali D., Vedi S., Compston J.E., Beresford J.N., Bhalla A.K. (2002). Methotrexate in the treatment of rheumatoid arthritis. II. In vivo effects on bone mineral density. Rheumatology.

[47] Poutoglidou F., Pourzitaki C., Manthou M.E., Samoladas E., Malliou F., Saitis A., Kouvelas D. (2021). Effects of Long-Term Methotrexate, Infliximab, and Tocilizumab Administration on Bone Microarchitecture and Tendon Morphology in Healthy Wistar Rats. Cureus.

[48] Satoh K., Mark H., Zachrisson P., Rydevik B., Byrod G., Kikuchi S., Konno S., Sekiguchi M. (2011). Effect of methotrexate on fracture healing. Fukushima J. Med. Sci..

[49] Carbone L.D., Kaeley G., McKown K.M., Cremer M., Palmieri G., Kaplan S. (1999). Effects of long-term administration of methotrexate on bone mineral density in rheumatoid arthritis. Calcif. Tissue Int..

[50] Tascioglu F., Oner C., Armagan O. (2003). The effect of low-dose methotrexate on bone mineral density in patients with early rheumatoid arthritis. Rheumatol. Int..

[51] di Munno O., Mazzantini M., Sinigaglia L., Bianchi G., Minisola G., Muratore M., la Corte R., di Matteo L., Canesi B., Caminiti M. (2004). Effect of low dose methotrexate on bone density in women with rheumatoid arthritis: Results from a multicenter cross-sectional study. J. Rheumatol..

[52] Bianchi M.L., Cimaz R., Galbiati E., Corona F., Cherubini R., Bardare M. (1999). Bone mass change during methotrexate treatment in patients with juvenile rheumatoid arthritis. Osteoporos. Int..

[53] Ozen G., Pedro S., Wolfe F., Michaud K. (2019). Medications associated with fracture risk in patients with rheumatoid arthritis. Ann. Rheum. Dis..

[54] Tronstad I., Hoff M., Horn J., Vikjord S.A.A., Videm V., Johansson J., Nilsen T.I.L., Langhammer A. (2024). Rheumatoid arthritis, disease-modifying antirheumatic drugs and risk of major osteoporotic fracture: Prospective data from the HUNT Study, Norway. RMD Open.

[55] Segawa Y., Yamaura M., Aota S., Omata T., Tuzuike N., Itokazu Y., Oka H., Tamaki H., Nakamura T. (1997). Methotrexate maintains bone mass by preventing both a decrease in bone formation and an increase in bone resorption in adjuvant-induced arthritic rats. Bone.

[56] Morgan S.L., Chen D.T., Carlee J., Baggott J.E. (2004). Effect of methotrexate therapy on bone mineral density and body composition in rat adjuvant arthritis. J. Rheumatol..

[57] Scheven B.A., van der Veen M.J., Damen C.A., Lafeber F.P., Van Rijn H.J., Bijlsma J.W., Duursma S.A. (1995). Effects of methotrexate on human osteoblasts in vitro: Modulation by 1,25-dihydroxyvitamin D3. J. Bone Miner. Res..

[58] Uehara R., Suzuki Y., Ichikawa Y. (2001). Methotrexate (MTX) inhibits osteoblastic differentiation in vitro: Possible mechanism of MTX osteopathy. J. Rheumatol..

[59] Annussek T., Kleinheinz J., Thomas S., Joos U., Wermker K. (2012). Short time administration of antirheumatic drugs—Methotrexate as a strong inhibitor of osteoblast’s proliferation in vitro. Head Face Med..

[60] Corrado A., Neve A., Marucci A., Gaudio A., Cantatore F.P. (2015). Combined effects of infliximab and methotrexate on rheumatoid arthritis osteoblastic cell metabolism. Clin. Exp. Med..

[61] May K.P., West S.G., McDermott M.T., Huffer W.E. (1994). The effect of low-dose methotrexate on bone metabolism and histomorphometry in rats. Arthritis Rheum..

[62] Annussek T., Szuwart T., Kleinheinz J., Koiky C., Wermker K. (2014). In vitro inhibition of HUVECs by low dose methotrexate—Insights into oral adverse events. Head Face Med..

[63] Hirata S., Matsubara T., Saura R., Tateishi H., Hirohata K. (1989). Inhibition of in vitro vascular endothelial cell proliferation and in vivo neovascularization by low-dose methotrexate. Arthritis Rheum..

[64] Yamasaki E., Soma Y., Kawa Y., Mizoguchi M. (2003). Methotrexate inhibits proliferation and regulation of the expression of intercellular adhesion molecule-1 and vascular cell adhesion molecule-1 by cultured human umbilical vein endothelial cells. Br. J. Dermatol..

[65] Marques C., Teixeira D., Cunha A., Meireles M., Pestana D., Keating E., Calhau C., Monteiro R., Faria A. (2013). Methotrexate enhances 3T3-L1 adipocytes hypertrophy. Cell Biol. Toxicol..

[66] Liu Y., Cui Y., Chen Y., Gao X., Su Y., Cui L. (2015). Effects of dexamethasone, celecoxib, and methotrexate on the histology and metabolism of bone tissue in healthy Sprague Dawley rats. Clin. Interv. Aging.

[67] von Brackel F.N., Grambeck J., Barvencik F., Amling M., Oheim R. (2024). MTX Osteopathy Versus Osteoporosis Including Response to Treatment Data-A Retrospective Single Center Study Including 172 Patients. Calcif. Tissue Int..

[68] Hauser B., Merriman A., Foley J., Golla J., McRorie E., Ralston S.H. (2025). Methotrexate continuation increases fracture risk in patients who sustained lower limb insufficiency fractures. Ann. Rheum. Dis..

[69] Robin F., Ghossan R., Mehsen-Cetre N., Triquet L., Larid G., Coiffier G., Mina M., Pickering M.E., Barthe C., Paccou J. (2025). METHOFRACT, a methotrexate osteopathy multicentre cohort study. RMD Open.

[70] Sokhan A., Haschka J., Messner Z., Kocijan R. (2025). MTX-Osteopathie in der rheumatologischen Praxis. Rheuma Plus.

[71] Yurtsever A., Fagerberg S.K., Rasmussen C. (2020). Insufficiency fractures of the knee, ankle, and foot in rheumatoid arthritis: A case series and case-control study. Eur. J. Rheumatol..

[72] Rozin A.P. (2003). Is methotrexate osteopathy a form of bone idiosyncrasy?. Ann. Rheum. Dis..

[73] Furudate K., Satake A., Narita N., Kobayashi W. (2018). Methotrexate-Related Lymphoproliferative Disorder in Patients with Osteonecrosis of the Jaw: A 3-Case Report and Literature Review. J. Oral Maxillofac. Surg..

[74] Henien M., Carey B., Hullah E., Sproat C., Patel V. (2017). Methotrexate-associated osteonecrosis of the jaw: A report of two cases. Oral Surg. Oral Med. Oral Pathol. Oral Radiol..

[75] Milosavljevic M., Jovanovic M., Folic M., Zivic M., Zdravkovic D., Velickovic S., Jankovic S. (2022). Possible association of methotrexate use with osteonecrosis of the jaw: Systematic review. J. Stomatol. Oral Maxillofac. Surg..

[76] Rolvien T., Creutzfeldt A.M., Lohse A.W., Amling M. (2019). Stress fractures in systemic lupus erythematosus after long-term MTX use successfully treated by MTX discontinuation and individualized bone-specific therapy. Lupus.

[77] Preston S.J., Diamond T., Scott A., Laurent M.R. (1993). Methotrexate osteopathy in rheumatic disease. Ann. Rheum. Dis..

[78] Tanaka K., Hashizume M., Mihara M., Yoshida H., Suzuki M., Matsumoto Y. (2014). Anti-interleukin-6 receptor antibody prevents systemic bone mass loss via reducing the number of osteoclast precursors in bone marrow in a collagen-induced arthritis model. Clin. Exp. Immunol..

[79] Llorente I., Garcia-Castaneda N., Valero C., Gonzalez-Alvaro I., Castaneda S. (2020). Osteoporosis in Rheumatoid Arthritis: Dangerous Liaisons. Front. Med..

